# MicroRNA-301a promotes migration and invasion by targeting TGFBR2 in human colorectal cancer

**DOI:** 10.1186/s13046-014-0113-6

**Published:** 2014-12-31

**Authors:** Wenpeng Zhang, Tao Zhang, Runsen Jin, Hongchao Zhao, Jin Hu, Bo Feng, Lu Zang, Minhua Zheng, Mingliang Wang

**Affiliations:** Department of General Surgery, Ruijin Hospital, Shanghai Jiao Tong University School of Medicine, Shanghai, 200025 P.R. China; ᅟ, Shanghai Minimally Invasive Surgery Center, Shanghai, 200025 P.R. China; Department of General Surgery, Luwan Branch of Ruijin Hospital, Shanghai Jiao Tong University School of Medicine, Shanghai, 200020 P.R. China; Department of General Surgery, the First Affiliated Hospital of Zhengzhou University, ᅟ, Zhengzhou, 450052 Henan Province China

**Keywords:** CRC, miRNA-301a, Metastasis, Invasion, TGFBR2

## Abstract

**Background:**

MicroRNAs (miRNAs) have been reported to play crucial roles in regulating a variety of genes pivotal for tumor metastasis. MicroRNA-301a (miR-301a) is overexpressed and displays oncogenic activity in many cancers. However, little is known about the potential roles of miR-301a in colorectal cancer (CRC).

**Methods:**

Taqman probe stem-loop real-time PCR was used to quantitatively measure the expression level of miR-301a in 48 cases of CRC tissues and the matched adjacent non-tumor mucosa as well as in CRC cell lines. miR-301a mimics and inhibitors were used to up-regulate and down-regulate miR-301a in CRC cells, respectively; lentivirus was used to construct miR-301a stably up- and down-regulated CRC cell lines. Metastasis ability was evaluated by transwell and wound healing assays while invasion was measured by transwell coated with matrix gel *in vitro*; *in vivo* metastasis was performed on nude mice model. The target of miR-301a was predicted by TargetScan software and validated by qRT-PCR, immunohistochemistry, western blot and luciferase reporter gene assay.

**Results:**

The expression of miR-301a was significantly higher in lymph node metastasis positive CRC samples compared with negative ones. Downregulation of miR-301a significantly inhibited the migration and invasion both *in vitro* and *in vivo* while forced up-regulation of miR-301a promoted migration and invasion. TGFBR2 was identified to be the downstream target of miR-301a. Knockdown of TGFBR2 in cells treated by miR-301a inhibitor elevated the previously abrogated migration and invasion.

**Conclusions:**

Our data indicated that miR-301a correlated with the metastatic and invasive ability in human colorectal cancers and miR-301a exerted its role as oncogene by targeting TGFBR2.

## Background

Colorectal cancer (CRC) is one of the most prevalent causes of cancer-related mortalities worldwide, with 610.000 deaths each year. Though efforts have been paid to the prevention of CRC, the incidence of CRC is still increasing in the past decades [1]. It has been reported that approximate 20% of CRC patients are diagnosed with metastases and 50% of early stage CRC patients treated will eventually develop metastases [2,3]. Despite advantages in early screening and development of new therapeutic strategies, the 5-year survival rate for patients with metastatic CRC is only about 10% [2]. Thus, an insight into the molecular mechanisms of CRC metastasis is important for developing new prognostic and therapeutic molecular markers to reduce CRC metastasis.

MicroRNAs (miRNAs) are a class of small non-coding RNAs (18–22 nt in length) which regulate the expression of target genes by binding to the 3’ un-translated regions (3’-UTR) [4,5]. Emerging evidences strongly suggest critical roles of miRNAs in the pathogenesis of cancer, and specially, the tumor metastasis [6,7]. The dysregulation of miRNAs in CRC has been reported using miRNA expression profiling studies with different miRNAs identified either as enhancers (miR-21, miR-31, miR-103, miR-107) or suppressors (miR-135, miR-145, miR-200c) in the initiation and evolution of tumor metastasis [8-13]. Considering the chemical stability, the ease of clinical detection, as well as the crucial roles of miRNA in tumor pathology, researches on the identification of potential miRNAs as metastatic biomarkers and therapeutic targets in CRC patients are rapidly increasing.

The gene encoding miR-301a is located in the human chromosome 17q22-17q23 and it has been previously reported to be overexpressed in many kinds of human cancers, including gastric cancer, liver cancer, breast cancer, etc. [14-20]. Besides, a series of recent functional studies demonstrated that miR-301a promoted cancer cell metastasis in breast, hepatocellular and gastric tumors through different target genes [17,21,22]. For CRCs, a miRNA array which analyzed the miRNA expression profiles of 44 pairs of CRCs revealed that the expression of miR-301a was elevated in tumor tissues of stage II patients compared to paired normal tissues [23]. However, until now, functional evidence of miR-301a in CRCs has not been documented and its role in CRC metastasis remains unclear. Thus, we wanted to know whether miR-301a was involved in the metastasis of CRC and we further wanted to evaluate the potential signalling pathways through which miR-301a regulated metastasis.

## Methods

### CRC samples

48 pairs of tumor samples and the paired adjacent non-tumor mucosa (at least 5 cm away from the tumor margin) were collected from CRC patients undergoing laparoscopic surgery from March 2011 to September 2011 at Shanghai Minimally Invasive Surgery Centre (Ruijin Hospital, Shanghai Jiao Tong University School of Medicine). The written informed consents were given by all patients prior to surgery and all procedures were approved by the ethics committee of Ruijin Hospital. None of the patients received preoperative chemoradiotherapy and all were diagnosed with resectable tumors by endoscopy and CT. All tumors were confirmed pathologically and classified according to the 7th edition of AJCC/UICC TNM system [24]. The clinicopathological information of included cases was prospectively collected during hospitalization. Samples were immediately excised and preserved in formalin for further immunohistochemical staining or snap-frozen in liquid nitrogen for future RNA extraction.

### Cell culture

Five human CRC cell lines were obtained from American Type Culture Collection (Manassas, United States). HCT116 and HT29 were cultured in Dulbecco’s modified Eagle’s medium (DMEM; Gibco, United States) with 10% fetal bovine serum (FBS). LOVO, SW480 and SW620 were maintained in RPMI 1640 (Sigma Aldrich, United States), supplemented with 10% FBS. All cells were maintained in a humidified incubator at 37°C with 5% CO_2_.

### RNA isolation and miRNAs assays

Total RNA was isolated from cell lines and tissue samples using Trizol reagent (Invitrogen, United States) according to the manufacturer’s instructions. The expression levels of miR-301a and U6 small nuclear RNA (RNU6B, Applied Biosystems, United States) were assayed in triplicates by the TaqMan stem-loop RT-PCR method with a mirVana miRNA detection Kit and gene-specific primers (Applied Biosystems, United States). Expression levels of RNU6B were used for normalization. The relative expression of miR-301a in tissues and cell lines were calculated by the 2^-Δct^ method.

### SYBR green real-time RT-PCR

The mRNA level of TGBR2 was measured by quantitative real-time polymerase chain reaction (qRT-PCR) with Power SYBR Green PCR Master Mix (Applied Biosystems, United States) in an Applied Biosystems 7500 System. The Super Script III First-Strand Synthesis System (Invitrogen, United States) was used to prepare cDNA from total RNA. The primers for TGFBR2 gene were obtained from “Primerbank” (http://pga.mgh.harvard.edu/primerbank/). The relative amount of TGFBR2 mRNA was uniformly normalized using glyceraldehyde 3-phosphate dehydrogenase (GAPDH) as endogenous control. Primer sequences are presented in Table 1.

**Table 1:** **Primer sequences used qRT-PCR**

	**Forward primer**	**Reverse primer**
TGFBR2	5′-GTAGCTCTGATGAGTGCAATGAC-3′	5′-CAGATATGGCAACTCCCAGTG-3′
GAPDH	5′-CGGAGTCAACGGATTTGGTCGTAT-3′	5′-AGCCTTCTCCATGGTGGTGAAGAC-3′

### Oligonucleotides transfection

miR-301a inhibitors (anti-miR-301a), miR-301a mimics and their corresponding negative controls (anti-miR-NC, miR-NC) were purchased from GenePharma (China, Table 2). siRNA of TGFBR2 was purchased from Dharmacon (D-003930, Dharmacon, United States). Cells were transfected with 100 nM oligonucleotides using Lipofectamine 2000 Reagent (Invitrogen, United States) according to the manufacturer’s instructions. 48 hours after transfection, cells were harvested and miR-301a expression level was monitored by qRT-PCR (U6 RNA was endogenous control), and the siRNA transfection efficiency was assessed by western blot using GAPDH as loading control.

**Table 2:** **The sequences of miR-301a inhibitor and mimics**

	**Sequences**
miR-301a-inhibitor	5′-GCUUUGACAAUACUAUUGCACUG-3′
Inhibitor NC	5′-CAGUACUUUUGUGUAGUACAA-3′
miR-301A-mimics	5′-CAGUGCAAUAGUAUUGUCAAAGCUUUGACAAUACUAUUGCACUGUU-3′
Mimics NC	5′-UAGCUAAGAGACCCAGAGUUAUUCUUACAAUCUCUCUACUAUC-3′

### Lentiviral transfection for stable expression clones

Plasmids LV3-pGLV-H1-GFP + Puro with hsa-miR-301a inhibitor or its negative control oligonucleotides, namely LV-anti-miR-301a and LV-anti-miR-301a-NC, were purchased from GenaPharma (China). The procedures to establish LV-anti-miR-301a stably expressing clones and the control clones in SW620 cells (SW620/LV-anti-miR-301a and SW620/LV-anti-miR-NC) were performed as previously described [25].

### Migration and invasion assays

Cell migration was performed using transwell inserts (Corning, United States), and cell invasion assay was measured using matrigel-coated upper chambers (BD Bioscience, United States). Briefly, cells (2.5 × 10^5^) in 200 μl serum-free medium were plated to the upper chambers and medium containing 10% serum was used as chemo attractant in lower chambers. After 24 hours incubation, non-migrating or non-invading cells were removed softly with cotton swabs and cells at the out surface of insert membranes were fixed with menthol for 30 min, stained with 1% crystal violet for another 30 min, and then photographed under 200 × magnification. Cells were counted from 5 random fields of view and means ± SD were calculated accordingly.

### Wound-healing assay

Generally, cells were first plated in a six-well plate and then transfected with different oligonucleotides. Forty-eight hours later, cells were harvested and plated in a new six-well plate. When the cells grew to a confluence of 90–95%, the cell monolayer was scratched using a sterile 10 μl pipette tip. After washing and removing the detached cells, the plates were incubated at 37°C for 24 h and the wounds were photographed. Then the open wound area was quantified using “TScratch” as previously described [26]. At least 5 different wounds were performed, and the experiments were repeated 3 times independently.

### *In vivo* metastasis assay

SW620/LV-anti-miR-301a and SW620/LV-anti-miR-NC cells (5 × 10^6^ cells per mice) were implanted subcutaneously into the left flanks of 5-week-old male nude mice (10 mice per group). Local invasion and lung metastasis were examined 4 or 7 weeks after implantation respectively (5 mice per group for each). Tumors and lungs were harvested, fixed, embedded and stained with haematoxylin and eosin as described previously [27]. All animal experiments complied with protocols approved by the Animal Care and Use Committee of Shanghai Jiao Tong University School of Medicine.

### Western blot analysis

Whole cell protein lysates were extracted using M-PER reagents and Halt Protease Inhibitor Cocktail kits (Pierce, United States). The protein concentrations were quantified with a Bicinchoninic Acid (BCA) protein assay kit (Pierce, United States). Western blotting for TGFBR2 was performed with established procedures as Ye et al. described previously [28]. The mouse monoclonal anti-TGFBR2 antibody (1:500, Abcam, United States) and anti-GAPDH antibody (1:2000, Kang Chen, China) were used as the primary antibodies. GAPDH served as loading control.

### Immunohistochemistry analysis

Immunohistochemistry analysis of TGFBR2 was performed with an anti-TGFBR2 antibody (1:200, Abcam, United States). Tumor tissues were fixed, embedded and stained with haematoxylin and eosin (Sigma, United States). Immunohistochemistry staining was performed with established protocols [29]. Relative TGFBR2 expression were defined as positive (moderate or strong staining) and negative (no or weak staining) based on the intensity of TGFBR2 staining of the tumor cells.

### Vector construction and luciferase reporter assay

miR-301a binding sites were predicted using TargetScan software (http://www.targetscan.org) and target genes which had the highest possibility and were also metastasis related were chosen for further validation. Bioinformatics analysis revealed two putative binding sites for miR-301a: a conserved 7mer-m8 at nt 266-272 of *TGFBR2* 3’-UTR and a poorly conserved 7mer-m8 at position 566-572 of *TGFBR2* 3’-UTR. Two mutant fragments of TGFBR2 3’-UTR (mut-266, mut-566) were designed using a Quick Change Site-Directed Mutagenesis kit (Stratagene, United States). Then the full length of miR-301a TGFBR2 3’-UTR including two wild-type binding sites and the two mutant fragments (mut-266, mut-566) were designed by and purchased from Sangon (Shanghai, China). After digestion by Sac I and Hind III, the fragments of wild-type and mutant were cloned into the Sac I and Hind III sites of pMIR-Report Luciferase Vector (Applied Biosystems, United States) and were named pMIR/TGFBR2-wt, pMIR/TGFBR2-mut-266 and pMIR/TGFBR2-mut-566, respectively. All constructs were verified by Sanger sequencing.

SW620 cells were co-transfected with 200 ng luciferase reporter gene construct, 2 ng pRL-TK vector (Promega, United States) containing Renilla luciferase, and 100 nM mimics or inhibitor in 24-well plates. Reporter assays were performed 48 h post-transfection using the Dual-luciferase assay system (Promega, United States). Firefly luciferase activity was normalized to renilla luciferase activity. All transfection experiments were conducted in triplicate and repeated 3 times independently.

### Statistical methods

Data were expressed as means ± standard deviation (SD) and *P <* 0.05 was considered statistically significant. Student’s t test (two-tailed) was used to compare two groups unless indicated specifically (χ^2^ test was used for comparison of the rates of two groups). Correlation analysis was performed with spearman correlation analysis. All statistical analyses were performed using the SPSS 15.0 software (SPSS Inc, United States).

## Results

### miR-301a is up-regulated in lymph node metastatic CRC tissues and CRC cells lines

**Figure 1:**
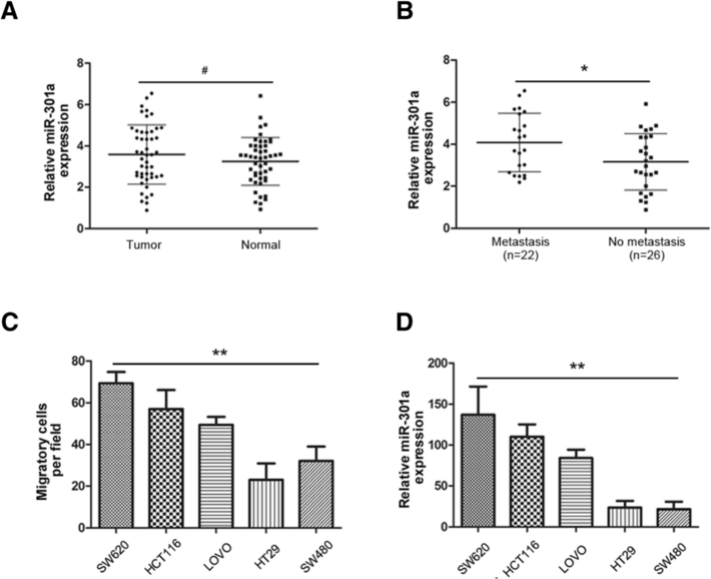
**miR-301a was upregulated in lymph node metastatic CRC tissues and highly metastatic CRC cells. (A)** qRT-PCR analysis of 48 pairs of CRC samples and matched adjacent normal mucosa; U6 small nuclear RNA was used as an internal control. There was no significant difference in miR-301a expression between CRC tissues and their adjacent non-tumor tissues. **(B)** miR-301a expression levels in primary CRCs with lymph node metastasis were much higher than that of CRCs without lymph node metastasis. **(C)** The migratory potential was examined by migration assays. **(D)** Relative expression of miR-301a in 5 CRC cell lines by qRT-PCR with U6 small nuclear RNA as an internal control. Data were representedas means ± SD from 3 independent experiments. ^#^, *P* > 0.05; *, *P* < 0.05; **, *P* < 0.01.

To understand the role of miR-301a in CRC progression, we examined the expression of miR-301a in 48 CRC tissues and their paired adjacent non-tumor tissues. qRT-PCR demonstrated that there was no significant difference of miR-301a expression between CRC and the adjacent non-tumor mucosa (Figure 1A, *P* > 0.05). However, the expression of miR-301a in tumors with lymph node metastases was significantly higher than that in tumors without metastases (Figure 1B, *P* < 0.05), which indicated that miR-301a might be involved in the metastasis of CRC. Importantly, the expression of miR-301a did not differ among patients with different gender, age, tumor differentiation, tumor location, tumor histopathology, tumor local invasion or distant metastasis (Table 3).

**Table 3:** **Clinicopathological parameters**

**Characteristics**	**No. of cases**	**miR-301 expression**	***P*** **-value**
Age(years)			
<66	20	3.76	
≥66	28	3.63	0.79
Sex			
Male	30	3.60	
Female	18	3.83	0.62
Tumor location			
Colon	23	3.27	
Rectum	25	4.07	0.08
Tumor invasion			
T_1_, T_2_	10	3.03	
T_3_, T_4_	38	3.86	0.14
Lymphatic metastasis			
Positive	26	3.16	
Negative	22	4.08	0.03
Distant metastasis			
M_0_	43	3.72	
M_1_	5	3.40	0.68
Differentiation			
Well and Moderate	34	3.91	
Poor	14	3.14	0.13

Next we wanted to know whether miR-301a was expressed in CRC cell lines with differential metastatic potential and whether the expression was associated with the metastatic ability. We conducted the migration assay with five CRC cell lines and found SW620 and HCT116 cells exhibited high metastatic ability while LOVO being the moderate and HT29 and SW480 as less metastatic cell lines (Figure 1C). Subsequently, we evaluated the expression of miR-301a in these cells and as shown in Figure 1D, the expression of miR-301a was much higher in SW620 and HCT116 than that of LOVO, HT29, and SW480. Specifically, expression of miR-301a seemed to be correlated with the metastatic ability of CRC cells by spearman correlation analysis (*r* = 0.96, *P* < 0.01). Altogether, these data indicated that miR-301a may contribute to the metastasis of CRC.

### miR-301a promotes CRC cell migration and invasion *in vitro*

**Figure 2:**
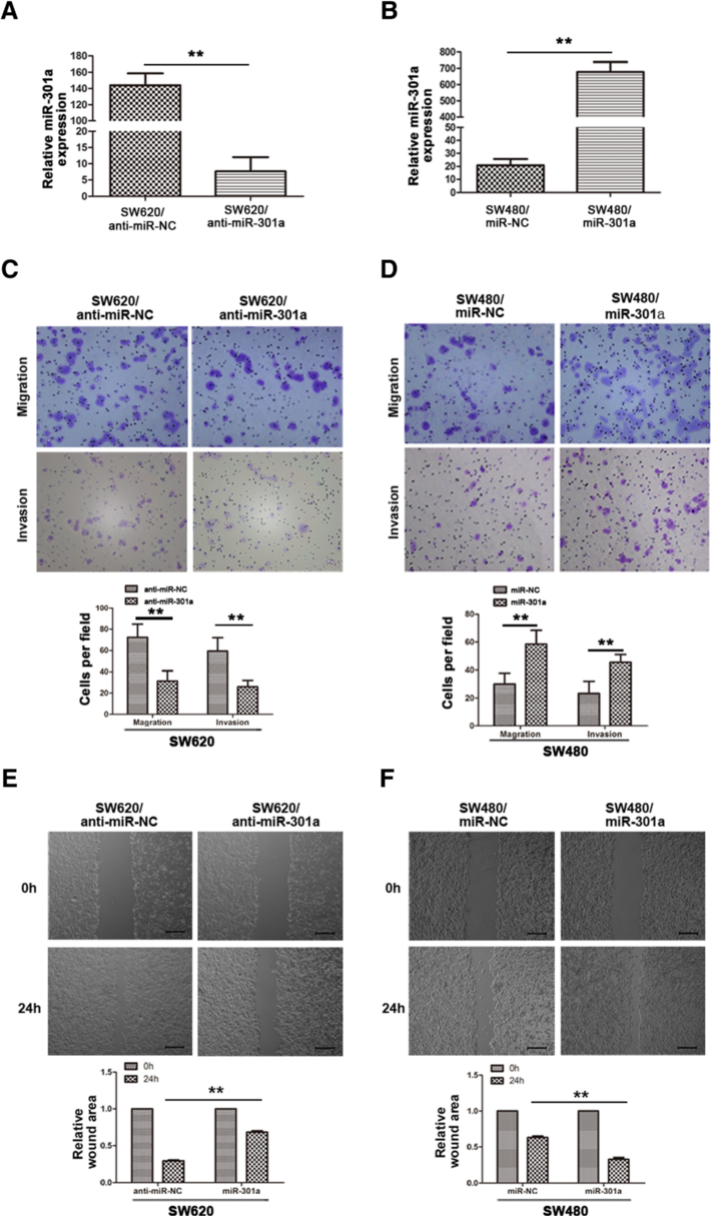
**miR-301a promoted CRC cell migration and invasion** ***in vitro*** **. (A)** Expression levels of miR-301a in SW620 cells after transfection with inhibitors of miR-301a, as determined by qRT-PCR. U6 small nuclear RNA was used as an internal control. **(B)** Expression levels of miR-301a in SW480 cells after transfection with synthetic miR-301a mimics, as determined by qRT-PCR. U6 small nuclear RNA was used as an internal control. **(C)** Downregulation of miR-301a with its inhibitors impeded cell migration and invasion of SW620 cells. **(D)** Overexpression of miR-301a significantly promoted cell migration and invasion in SW480 cells. **(E)** miR-301a inhibitors significantly inhibited SW620 cell motility as shown in wound healing assay. **(F)** miR-301a mimics significantly promoted SW480 cell migration in wound healing assay. Data were representedas means ± SD from 3 independent experiments. **, *P* < 0.01.

To further determine whether miR-301a could affect CRC cell migration, we transfected SW620 cells, which showed a high level of miR-301a, with the anti-miR-301a in order to suppress miR-301a (i.e. SW620/anti-miR-301a cells). Meanwhile, anti-miR-control was transfected as negative control (i.e. SW620/anti-miR-NC cells). Transient transfection of anti-miR-301a in SW620 cells significantly suppressed the expression of miR-301a in SW620/anti-miR-301a cells (Figure 2A*, P* < 0.01). Next we conducted cell migration assay to evaluate whether miR-301a could influence metastasis directly. As shown in Figure 2C, SW620/anti-miR-301a cells showed a significant (*P* < 0.01) reduction of migratory ability, while, unexpectedly, the invasive capacity was also significantly (*P* < 0.01) decreased. To further support the role of miR-301a in migration, we performed the wound-healing assays to explore cell motility change. In consistent with the migratory change, SW620/anti-miR301a cells exhibited significant lower migratory potential compared with the control cells (Figure 2E, *P* < 0.01).

Additionally, the gain-of-function assay with miR-301a mimics transfected in miR-301a low-expressed SW480 cells was performed to further consolidate the role of miR-301a in cell migration and invasion. As shown in Figure 2B, exogenous expression of miR-301a in SW480 cells resulted in a significant increase of miR-301a by qRT-PCR (*P* < 0.01). Upregulation of miR-301a in SW480 cells led to a marked increase of both migratory and invasive abilities in contrast with the control cells (Figure 2D, *P* < 0.01). Consistently, SW480 cells transfected with miR-301a mimics migrated much faster than the control cells in the wound-healing assay (Figure 2F, *P* < 0.01). Conclusively, miR-301a promotes the migration and invasion ability of CRC cells *in vitro*.

### miR-301a promotes tumor metastasis and cell invasion *in vivo*

**Figure 3:**
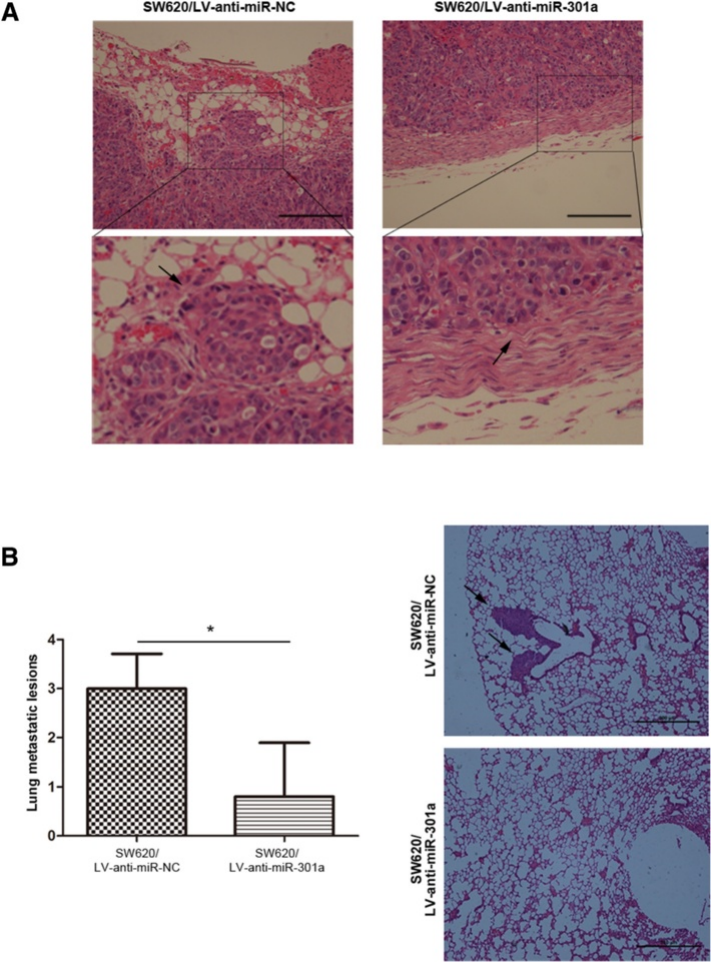
**Downregualtion of miR-301a suppressed CRC cell invasion and lung metastases** ***in vivo*** **. (A)** miR-301a inhibited CRC cell invasion. H&E staining of tumor acquied from nude mice bearing the SW620/LV-anti-miR-301a or SW620/LV-anti-miR-NC cells (indicated by arrows), 4 weeks post implantation. **(B)** Mean lung metastatic nodules (left) and histologic analysis (H&E staining) of lung (right) 7 weeks post implantation. Metastatic tumor lesions are indicated by arrows. Data were represented as means ± SD. Magnification, ×200. Scale bars, 100 μm. *, *P* < 0.05.

The above mentioned results further led us to ask whether miR-301a functioned as a promoter of migration and invasion of CRC cells *in vivo* as well. To this end, we constructed miR-301a stably down-regulated and negative control SW620 cells with lentivirus particles LV-anti-miR-301a and LV-anti-miR-301a-NC, respectively. SW620/LV-anti-miR-301a cells and SW620/LV-anti-miR-NC cells were separately injected into nude mice subcutaneously and mice were sacrificed four weeks later. Tumors were processed and stained with H&E staining. As shown in Figure 3A, tumors grown of SW620/LV-anti-miR-301a cells were less-invasive as most tumors (4/5) confined within the fibrous capsules without breaking into the stromal (Figure 3A, right panel). In contrast, tumors of the control group had cancer nests formed by SW620/LV-anti-miR-NC cells which invaded the adjacent stromal tissues (Figure 3A, left panel).

To validate the role of miR-301a in metastasis *in vivo*, we dissected the lung of nude mice and evaluated the metastatic sites both micro- and macroscopically. In line with the *in vitro* results, fewer metastatic lesions in the lung of nude mice injected with SW620/LV-anti-miR-301a were seen compared with that of SW620/LV-anti-miR-NC group (Figure 3B; *P* < 0.05), indicating the suppression of metastatic ability in SW620/LV-anti-miR-301a cells *in vivo*. Hence, miR-301a could promote tumor metastasis and invasion *in vivo*.

### miR-301a represses TGFBR2 protein expression by binding to 3’-UTR

It’s well-established that miRNAs exert their biological functions by binding to the 3’-UTR and inhibiting the translation of their target genes. Thus we wanted to know the target through which miR-301a exerted its function in CRC cells. For this reason, we used bioinformatics method to predict the potential targets of miR-301a. By searching the online TargetScan and PicTar algorithms systems, we identified transforming growth factor receptor 2 (TGFBR2) as the target of miR-301a with the highest possibility.

TGFBR2 is a major trans-membrane receptor of transforming growth factor-β (TGF-β). Upon binding with its ligand TGF-β, TGFBR2 is phosphorylated at the serine and threonine residues within its GS box, which could activate the TGF-β signaling pathway and lead to acquisition of resistance to the anti-mitogenic effects of TGF-β and contribute to tumor development and progression [30,31].

**Figure 4:**
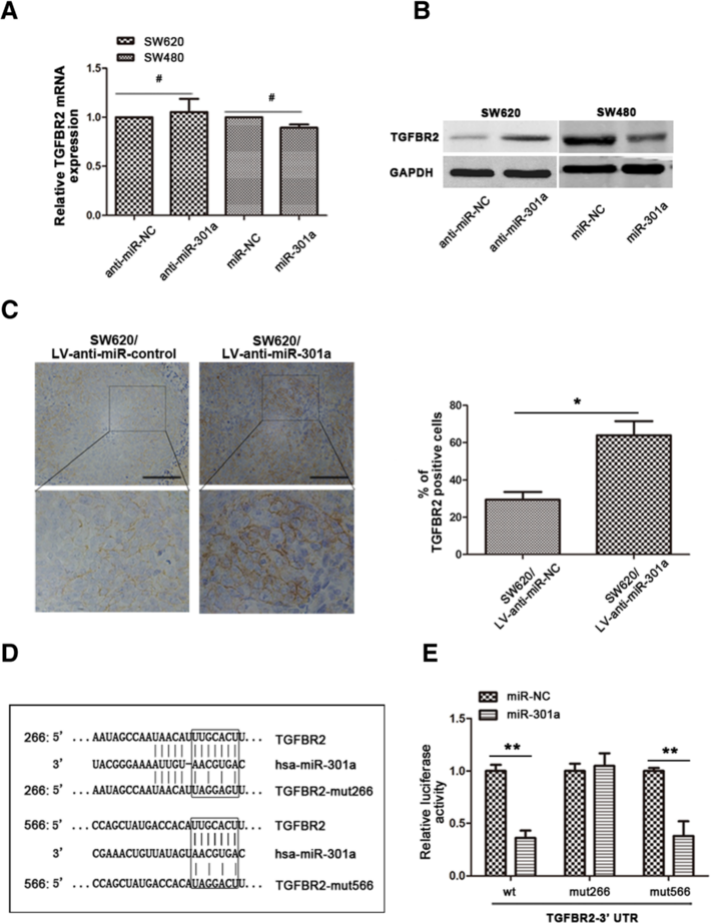
**TGFBR2 was a direct target gene of miR-301a. (A)** qRT-PCR showed that neither miR-301a mimics nor anti-miR-301a changed the mRNA level of TGFBR2. **(B)** anti-miR-301a in SW620 cells suppressed, while miR-301a mimics in SW480 cells increased the protein expression of TGFBR2. **(C)** Immunohistochemical staining of TGFBR2 in tumor sections. Expression of TGFBR2 was evaluated in tumors formed by SW620/LV-anti-miR-301a cells. **(D)** Schematic picture of two miR-301a binding sites and the corresponding mutants on TGFBR2 3’-UTR. **(E)** Luciferase activity assay identified the binding sites of miR-301a. Data were representedas means ± SD from 3 independent experiments. Magnification, ×200. Scale bars, 100 μm. *, *P* < 0.05; **, *P* < 0.01.

Thus, we attempted to figure out whether TGFBR2 was a downstream target of miR-301a or not. qRT-PCR and western blot analysis were first employed and somehow unexpectedly, there was no significant difference of *TGFBR2* mRNA levels between SW620/anti-miR-301a and SW620/anti-miR-NC cells or SW480/miR-301a and SW480/miR-NC cells (Figure 4A). However, western blot demonstrated that expression of TGFBR2 protein was markedly increased in miR-301a downregulated SW620 cells and significantly reduced in miR-301a overexpressed SW480 cells (Figure 4B). This suggested that miR-301a suppressed TGFBR2 by post-transcriptional mechanism rather than directly inhibiting the transcription. Besides, suppression of TGBFR2 by miR-301a was also validated *in vivo*: in the xenograft tumors from SW620/LV-anti-miR-301a nude mice, staining intensity of TGFBR2 elevated remarkably compared to that of the SW620/LV-anti-miR-NC tumors (Figure 4C). Combined together, these results suggested that miR-301a suppressed TGFBR2 in CRC cells.

We then wanted to know the mechanism through which miR-301a regulated TGFBR2. In the 3’-UTR of *TGRBR2* mRNA there existed two putative binding sites for miR-301a: a highly conserved 7mer-m8 at nt 266-272 and a poorly conserved 7mer-m8 at position 566-572 (Figure 4D). To clarify whether miR-301a could interact with the 3’-UTR of TGFBR2, we performed luciferase reporter assays. Wild type and two mutant fragments of 3’-UTR were co-transfected with miR-301a mimics or NC, respectively. As shown in Figure 4E, compared to the miR-NC, miR-301a mimics significantly (*P* < 0.01) reduced the relative luciferase activity of pMIR/TGFBR2-wt and pMIR/TGFBR2-mut566 of TGFBR2. However, miR-301a did not affect the relative luciferase activity of the pMIR/TGFBR2-mut266, suggesting that miR-301a specifically bind to the conserved 7mer-m8 at nt 266-272 on 3’-UTR of *TGFBR2*. These data demonstrated unambiguously that miR-301a suppressed TGFBR2 protein expression *via* targeting its specific RNA binding site.

### TGFBR2 is a functional target through which miR-301a regulated metastasis and invasion in CRC cells

**Figure 5:**
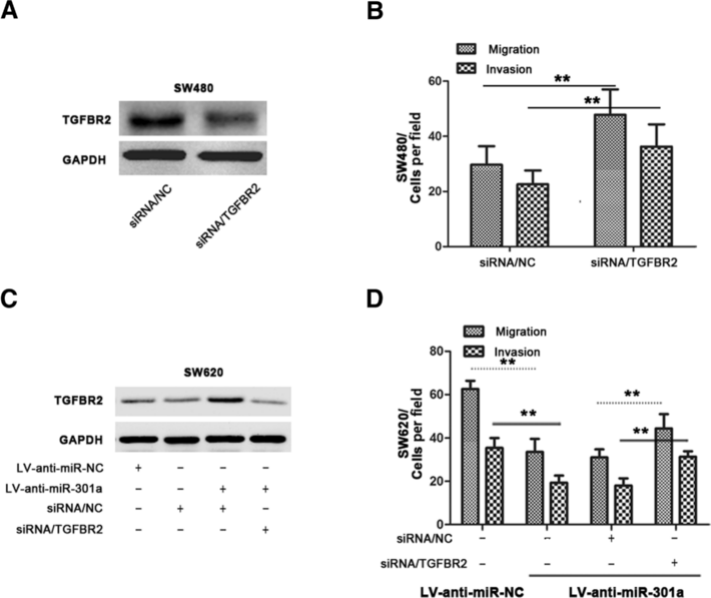
**miR-301a regulated metastasis and invasion through suppressing TGFBR2 in CRC cells. (A)** Western bloting showed TGFBR2 was downregulated in SW480 cells transiently transfected with TGFBR2 siRNA. **(B)** The cell invasion and migration ability were improved in TGFBR2 downregulated SW480 cells. **(C)** Expression of TGFBR2 protein in SW620/LV-anti-miR-301a or SW620/LV-anti-miR-NC cells transiently transfected with TGFBR2 siRNA or siRNA/NC. **(D)** Cell invasion and migration assays of SW620/LV-anti-miR-301a or SW620/LV-anti-miR-NC cells transiently transfected with TGFBR2 siRNA or siRNA/NC. Data were representedas means ± SD from 3 independent experiments. **, *P* < 0.01.

The results presented above confirmed TGFBR2 as one downstream target of miR-301a; however, it remained unclear whether miR-301a regulated metastasis and invasion in CRC cells through modulating TGFBR2. To this end, we focused on whether artificial expression or suppression of TGFBR2 protein could offset the effects caused by miR-301a mimics or anti-miR-301a, respectively. First, siRNA targeting TGFBR2 was transiently transfected into SW480 cells and as shown in Figure 5A, expression of TGFBR2 was markedly suppressed and this resulted in enhanced migration and invasion in SW480 cells (Figure 5B, *P* < 0.01), suggesting the involvement of TGFBR2 in CRC cell metastasis and invasion.

**Figure 6:**
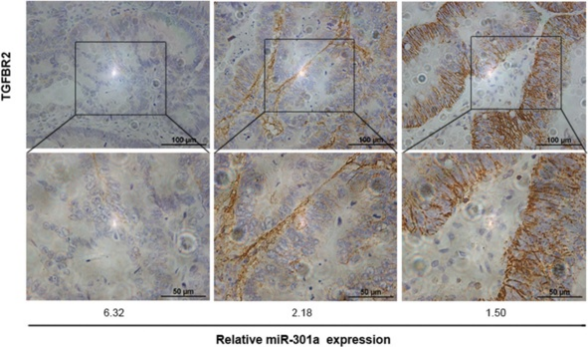
**Immunohistochemical staining of TGFBR2 in CRCs.** Higher expression of TGFBR2 was observed mainly in CRCs with low miR-301a level (*r* = -0.72, *P* < 0.001). Magnification, ×200. Scale bars, 100 μm.

To further determine whether TGFBR2 is responsible for the migration and invasion caused by miR-301a, we transfected TGFBR2 siRNA into SW620/LV-anti-miR-301a cells transiently. As shown in Figure 5C, the enhanced TGFBR2 level by LV-anti-miR-301a was depleted upon TGFBR2 siRNA transfection. Remarkably, compared with the SW620/LV-anti-miR-301a cells treated with siRNA/NC, SW620/LV-anti-miR-301a cells transfected with TGFBR2 siRNA regained the previously abrogated migration and invasion ability (Figure 5D, *P* < 0.01). Additionally, we evaluated the expression of TGFBR2 in CRCs. The archived tumors of the included 48 cases were retrieved and stained. As shown in Figure 6, higher TGFBR2 staining positivity was seen in tumors with low miR-301a expression; furthermore, we quantified the TGFBR2 staining intensity and made a correlation analysis between TGFBR2 and miR-301a. Expression of TGFBR2 was significantly and inversely associated with the expression of miR-301a (*r* = -0.72, *P* < 0.001). Conclusively, these results suggest that downregulation of TGFBR2 is involved in miR-301a-induced metastasis and invasion; TGFBR2 is a functional target of miR-301a.

## Discussion

Cancer metastasis is a complex, multi-step process which includes intricate cascades of molecular events. It’s well-established that miRNAs are involved in CRC tumorigenesis and many of them can serve as activators or suppressors of CRC metastasis [8-10,13]. miR-301a, one micro-RNA reported to be expressed at different levels in a variety of cancers, has been demonstrated to be important in promoting cancer metastasis [17,21,22]. However, the expression of miR-301a in CRCs and its role in CRC metastasis remain unclear. In the current study, we reported for the first time that miR-301a was up-regulated in lymph node metastatic CRC tissues and promoted CRC metastasis both *in vitro* and *in vivo*. Furthermore, we identified TGFBR2 to be one downstream target of miR-301a and also the effector of miR-301a in the regulation of metastasis in CRC cells.

miR-301a has been discovered up-regulated in various types of human cancers and proved to be an oncogene in gastric cancer [22], pancreatic cancer [32], hepatocellular cancer [21], and esophageal cancer [33]. In this study, we investigated the expression of miR-301a in CRC specimens for the first time. Unlike its expressions in the cancers mentioned above, there is no statistical difference of miR-301a expression between CRC tissues and their adjacent non-tumor tissues, nevertheless, increased miR-301a expression was observed in tumors with lymph node metastasis. This was in consistent with its role as an oncogene in aforementioned cancers and might indicate a metastasis promotion role rather than a tumor initiation role of miR-301a in CRCs. In supporting this, we found CRC cell lines with high metastasis capacity expressed higher miR-301a than those with low metastasis capacity and we went a step further by showing that miR-301a expression was correlated positively with the cell migratory ability in these CRC cells. These data led us to ask whether miR-301a could regulate metastasis in CRCs or not.

Actually, previous studies have shown that miR-301a acted as a metastatic promoter in different kinds of human cancers. Shi et al first reported the expression of miR-301a in human cancer: miR-301a overexpression has been implicated as a negative prognostic indicator in lymph node negative invasive ductal breast cancer and strongly associated with tumor recurrence. Besides knockdown of miR-301a reduced migration and invasion of both MCF-7 and MDA-MB-231 breast cancer cell lines *in vitro* [17]. Likewise, a recent study described that the expression level of miR-301a was significantly elevated in the primary tumors of 10 breast cancer patients with distant metastasis, as compared with that in primary tumors from 10 patients without detectable distant metastasis [34]. Additionally, overexpression of miR-301a in breast cancer cell lines could promote cell migration and invasion *in vitro* and lung metastases in mice models. Moreover, downregulation of miR-301a in hepatocellular carcinoma (HCC) cells suppressed invasion and migration of HCC cells *in vitro* [21]. In gastric cancer cells, ectopic expression of miR-301a led to significantly enhanced cell migration and invasion, whereas knockdown of miR-301a also inhibited cell metastasis [22].

In line with these findings, we demonstrated that overexpression of miR-301a in less aggressive SW480 cells led to a significant increase of cell migration whereas downregulation of miR-301a in more aggressive SW620 cells resulted in decreased migratic ability, indicating the metastasis promoter role of miR-301a in CRC. Furthermore, this effect was also prominent *in vivo*: knockdown of miR-301a significantly reduced xenograft tumor invasion and lung metastases in nude mice. These results indicated that miR-301a is a metastatic promoter in CRC.

miRNAs exert their biological functions by binding with the 3’-UTR of their target genes. Thus, identifying the downstream target genes could help understand miRNA physiological and pathological roles. Though several approaches have been developed to search miRNA targets in recent years, the computational approach, a method based on the principle that all known miRNA targets have conserved perfect or near-perfect complementary sites for miRNAs, was a powerful and prevalently used strategy [35-37]. Thus, we used the online TargetScan and PicTar algorithms systems to search for potential targets of miR-301a. As a result, TGFBR2, a gene previously reported involved in metastasis, was identified to be the target with highest possibility.

TGFBR2, a trans-membrane serine-threonine kinase, is a major member of TGF-β signaling and often found to be altered in CRCs [38]. The TGF-β signaling pathway plays a complex role in several CRC biological processes, including cell differentiation, proliferation, apoptosis, and motility [39]. It is commonly speculated that CRCs acquire partial TGF-β resistance largely because of TGFBR2 inactivation. Importantly, TGFBR2 is a metastatic suppressor in CRC: previous research showed that downregulation of TGFBR2 increased migration and invasion of HT29 cells *in vitro* [27] whereas evidences from *in vivo* studies suggested that loss of TGFBR2 contributed to colon cancer development and metastasis in cooperation with mutant K-ras or in the context of PTEN loss [40,41]. Indeed we found TGFBR2 was one target of miR-301a and the luciferase reporter gene assay successfully identified the binding site for miR-301a on the 3’-UTR of *TGFBR2* gene. In consistent with this, we found knockdown of miR-301a in CRC cells upregulated the protein level of TGFBR2 while ectopically overexpression of miR-301a suppressed its expression. Supporting this, we observed an inverse correlation between miR-301a and TGFBR2 protein expression in CRC tumor tissues. Functionally, we found TGFBR2 acted as a metastasis suppressor and also the effector of miR-301a in CRC cells: knockdown of TGFBR2 induced a significant increase in motility and invasiveness of SW480 cells whereas downregulation of TGFBR2 also elevated the previously abrogated migration and invasion ability initiated by LV-anti-miR-301a in SW620 cells. Taken together, TGFBR2 played an important role in miR-301a mediated tumor migration and invasion in CRC.

Interestingly, we found miR-301a regulated TGFBR2 expression by some translational suppression mechanisms: the mRNA level of TGFBR2 did not change significantly upon transfection with miR-301a inhibitor or mimics while the protein level showed remarkable differences. It’s proposed that miRNAs exert their functions in two ways: when an miRNA perfectly or near-perfectly pairs with its target mRNAs, it was thought that mRNA cleavage is the primary mechanism for miRNA mediated gene regulation; in case of a miRNA imperfectly pairs with its target mRNAs, translational suppression is thought to happen with protein expression downregulated while mRNA level remained stable [42,43]. In this study, though we found miR-301a indeed bound with TGFBR2, they might not perfectly match in sequence since we observed marked downregulation of TGFBR2 protein while the mRNA of TGFBR2 remained unchanged. Thus we suggest that miR-301a imperfectly bind with the 3’-UTR of *TGFBR2* and regulate the expression of TGFBR2 via translational suppression.

## Conclusions

In summary, our study presented convincing evidences to show that miR-301a exerted its role as oncogene in CRC metastasis, and for the first time we identified TGFBR2 as a functional target involved in miR-301a modulated CRC cell metastasis. miR-301a could be regarded as a new target for CRC prevention and therapy.

## Abbreviations

miRNAs: microRNAs
miR-301a: microRNA-301a
CRC: Colorectal cancer
TGFBR2: Transforming growth factor receptor 2
3’-UTR: 3’ un-translated regions
qRT-PCR: Quantitative real-time polymerase chain reaction
GAPDH: Glyceraldehyde-3-phosphate dehydrogenase
ATCC: American Type Culture Collection
cDNA: Complementary DNA
nt: Nucleotide
FBS: Fetal Bovine Serum
NC: Negative control
HE: Hematoxylin-eosin
FBS: Fetal bovine serum
DMEM: Dulbecco’s modified Eagle’s medium
RNU6B: U6 small nuclear RNA
HCC: Hepatocellular carcinoma
PTEN: Phosphatase and tensin homologue on chromosome 10
